# Effect of Nuts on Gastrointestinal Health

**DOI:** 10.3390/nu15071733

**Published:** 2023-04-01

**Authors:** Giuseppina Mandalari, Teresa Gervasi, Daniel W. Rosenberg, Karen G. Lapsley, David J. Baer

**Affiliations:** 1 Department of Chemical, Biological, Pharmaceutical and Environmental Science, University of Messina, Viale Ferdinando Stagno d’Alcontres 31, 98166 Messina, Italy; 2 Department of Biomedical and Dental Sciences and Morphofunctional Imaging, University of Messina, 98125 Messina, Italy; 3 Centre for Molecular Oncology, University of Connecticut Health Center, Farmington, CT 06030-3101, USA; 4 The Almond Board of California, Modesto, CA 94587, USA; 5 USDA, Agricultural Research Service, Beltsville Human Nutrition Research Center, Beltsville, MD 20705, USA

**Keywords:** nuts, gut health, microbiota, digestion

## Abstract

Nuts are high nutrient-dense foods containing healthy lipids, dietary fiber, and bioactive phytochemicals, including vitamins and minerals. Although the beneficial effect of nut consumption on different chronic diseases has been well documented, especially in relation to their cardiometabolic benefits, less scientific evidence is available on their possible beneficial effects on gastrointestinal health. In this narrative review, we summarize the most important findings and new research perspectives in relation to the importance of nut consumption on gastrointestinal health. The integrity of the cell wall structure, cell size and particle size after mastication are known to play a crucial role in energy, nutrient and bioactive release from nuts during digestion, therefore affecting bioaccessibility. Other mechanisms, such as cell wall composition, thickness and porosity, as well as stability of the membranes surrounding the oil bodies within the cell, are also important for energy extraction. As the undigested nutrients and phytochemicals are delivered to the colon, effects on gut microbiota composition are predicted. Although the overall effect of nut consumption on microbial alpha- and beta-diversity has been inconsistent, some scientific evidence suggests an increase in fecal butyrate after almond consumption, and a beneficial role of walnuts on the prevention of ulcerative colitis and protection against the development of gastric mucosal lesions.

## 1. Introduction

Nuts, including peanuts, are nutrient dense foods containing healthy lipids, beneficial phytonutrients and a range of essential vitamins and minerals [1,2]. After cereals, nuts are the plant food group highest in dietary fiber, which results in unique microstructure and physical properties. Since nuts resist digestion in the upper GI tract, their cellular structure retains intact lipids and polymerized polyphenols and plays a key role in how they are metabolized by gut microbiota in the colon to form bioactive molecules which could benefit human health [3]. The role for specific foods and dietary patterns in modifying gut microbiota and fecal metabolites and their impact on various aspects of human health is well known. Research to understand the composition and function of the microbiota has expanded dramatically in recent years with the development of increasingly sensitive analytical techniques. These tools have facilitated data mining to better understanding the relationship of the microbiome to physiology and health [4].

There are now four tree nuts (almond, cashews, pistachios, walnuts) for which human clinical trials have clearly shown that the measured (metabolizable) energy value is 5–25% lower that the calculated values used in food labelling [5]. Considering these nuts have varying cellular structures and macro, micro, and phytonutrient contents, the mechanisms for digestion and microbiota changes are not fully understood, although the evidence for lipid encapsulation is compelling [6]. Some studies have shown that an optimized diet rich in nuts may be an intervention that promotes a healthy microbial population and thereby improves overall physiology, but clinical trials to date are inconclusive.

In 2020, a systematic review [7] and meta-analysis [8] of a total of 10 randomized, controlled trials (RCTs) assessed the effects of various nuts on fecal microbiota for over 600 adults consuming western diets, with 40–100 g nuts daily, in the U.S.A., Germany, Italy and Spain. Fitzgerald et al. [7] concluded from nine RCTs (four almond, three walnut, one each hazelnut and pistachio) that the overall gut health benefits of nuts may be due, in part, to their unique composition and physical structure. However, the exact mechanisms by which nuts exert these modest modulatory effects on gut microflora remain unclear. Since specific microbial alterations were evident, but often inconsistent, the authors recommended future studies designed to address the baseline habitual dietary patterns and microbial composition to minimize inter-individual composition of the gut microflora. Creedon et al. [8] found the strength of evidence from their meta-analysis from nine RCTs (five almond, three walnut and one pistachio) to be generally inconclusive. Nut consumption affected gut microbiota composition at the genus level, but not at a phyla level nor on the diversity of the microbiome. However, nut type and, to some extent, their duration of consumption influenced the overall effects. They concluded that further parallel design RCTs, powered to detect changes in fecal microbiota and that incorporate functional and clinical outcomes, are still needed. Mead et al. [9] performed a systematic review of four studies that included children between the ages of 3 and 18 years (one almond, two hazelnut, one Brazil nut) who consumed between 15–30 g nuts daily for 8–16 weeks. Although they found nut consumption improved overall diet quality in this young population, there were inconsistent effects on gut health. They concluded that further studies were needed, with consideration given to higher doses and longer intervention periods.

Dietary pattern analysis has emerged as an alternative approach to study the relation between nutrition and disease. Nuts are typically included in different healthy food patterns, and, as part of the Mediterranean Diet (MedDiet), a dietary pattern widely recognized as a nutritional strategy that improves cardiometabolic health [10]. In Spain, Galie et al. [11] examined whether following a MedDiet modified gut microbiota composition and fecal metabolomics profiles, as well as cardiometabolic risk factors, compared with a non-MedDiet supplemented with 50 g nuts daily. They reported for the first time that the 50 participants with metabolic syndrome following the MedDiet, compared with a non-MedDiet diet supplemented with nuts, significantly changed specific microbial genera and fecal metabolites. However, it was concluded that further intervention studies were needed to understand the effects of different healthy dietary patterns on gut microbiota composition and functionality. In a separate study, Israeli researchers used a different approach to augment the MedDiet. In these studies, conducted in Spain and Italy, Rinott et al. [12] showed that within a 6 month controlled-feeding trial of 294 subjects, a green MedDiet, that included 28 g walnuts per day as well daily polyphenol-rich green tea and Mankai aquatic plant, led to more prominent compositional change in the gut microbiota.

They found both MedDiets induced substantial changes to the community structure of the gut microbiome, with the green MedDiet leading to more prominent compositional changes, largely driven by the low-abundant, “non-core”, microorganisms [12]. They concluded that the diet microbiome–host interaction should be further explored in future studies that may guide the implementation of novel beneficial modifications of existing dietary patterns.

An overview on the digestibility of nut nutrients and phytochemicals and the impact of food matrices and processing on digestion in the upper gastrointestinal (GI) tract is provided. In addition, the effect of nuts on the composition and diversity of the gut microbiota and their impact on the production of microbially-derived short chain fatty acids and bile acids, as well as recent reports describing the prevention of gastrointestinal diseases associated with nut consumption, is described in this review.

## 2. Food Matrix and Digestion

### 2.1. Microstructure and Cell Properties

**Figure 1:**
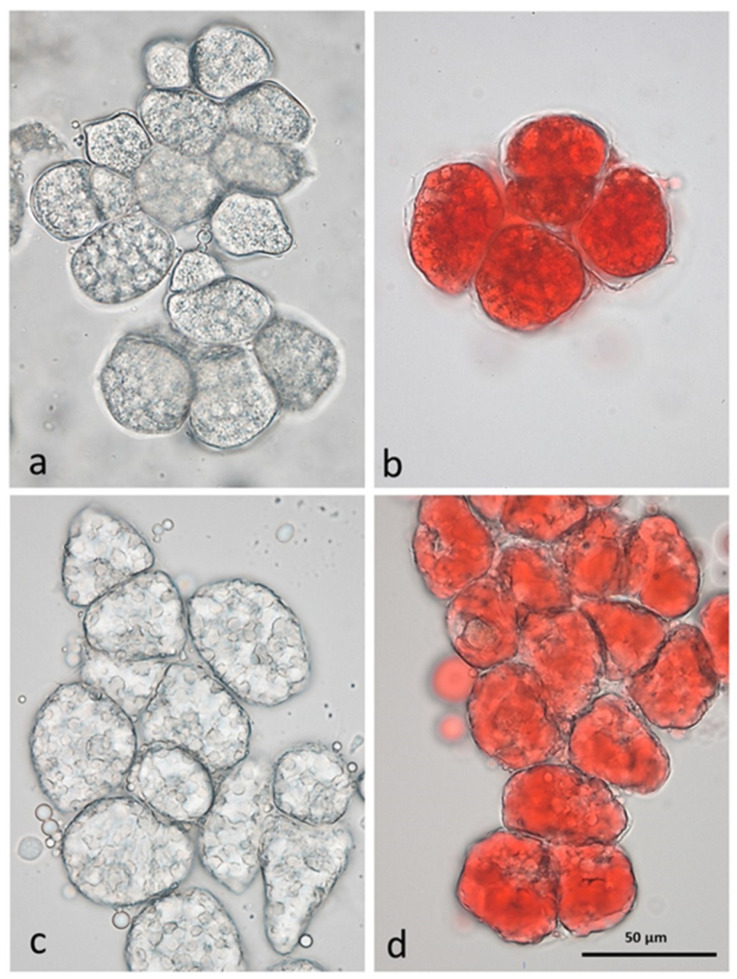
CDTA-separated cells of baseline natural raw almonds (**a**) unstained, (**b**) lipid stained with Sudan IV; and roasted almonds (**c**) unstained, (**d**) lipid stained with Sudan IV, showing lipid coalescence in the cells following roasting [14].

The great diversity of species, as well as of varieties within the same species, the cultivation methods, and the climatic characteristics where tree nuts are cultured, combine to exert profound effects on the chemical composition of nuts. The geometrical properties of nut shells and kernels, including length, width, thickness, density, surface area, volume, and specific gravity, influence the quality of nut products in the post-harvest process. Nut structure can influence lipid digestibility [13]. We have previously demonstrated that individual raw almond cells separated by the chelating agent CDTA are small (less than 50 µm in diameter) and the lipid is still within oleosomes (Figure 1), surrounding the protein bodies [14]. Roasting has an effect liberating the lipid from the oil bodies, which will then form large lipid droplets in unstained and Sudan IV-stained cells.

The physicochemical properties of the cell walls (e.g., dietary fiber), as well as their composition, mainly comprised of non-starch polysaccharides, are factors known to influence nutrient digestibility [15]. In addition, phospholipids and proteins can also limit the access of hydrolytic enzymes.

A theoretical model has been developed to predict lipid release from almonds in the gut: using simple geometry and data on cell dimensions and particle size, the number of ruptured cells in cut almond cubes was calculated [16]. The model has the potential to accurately predict nutrient bioaccessibility in a broad range of edible plants, based on their particle size and cell diameter. Grassby et al. [17] have also demonstrated that test meals containing almonds of different particle sizes behaved differently in the gut. Using a theoretical model, Creedon et al. [18] revealed a greater lipid bioaccessibility for ground almonds than whole almonds after mastication (10.4% ± 1.8% vs. 9.3% ± 2.0%, respectively; *p* = 0.017).

### 2.2. Bioaccessibility of Nutrients and Phytochemicals in the Upper Gastrointestinal Tract

During the multistage processing that occurs in the digestive system, mechanical and chemical mechanisms promote the breakdown of food molecules into smaller moieties, which can then be absorbed by the body. With the term “bioaccessibility”, we refer to the proportion of nutrients and/or phytochemicals released from the upper GI tract, thereby becoming potentially available for absorption [19]. The physicochemical properties of nuts significantly affect the bioaccessibility of their constituent nutrients and phytochemicals [20,21].

#### 2.2.1. Nutrient Bioaccessibility

Studies on almond digestion have shown that mechanical trituration or chewing breaks down a large fraction of the first outer layer of cells, while the majority of parenchyma cells, in which lipids and proteins are encapsulated, remains intact [19,22]. In a study with ileostomy volunteers, we showed that the lipids present in the intact cells located under the fractured layers appeared to ‘leach’ from the intact cells only after a protracted incubation in the upper GI tract [23]. This may be due to the increased porosity of the cells and to the degradation and solubilization of pectic compounds present in the cell wall and middle lamella [24]. The fractured surface may account for the lipid release that occurs after prolonged incubation in the GI tract. Although it is unclear to what extent lipolysis occurs inside almond cells and whether the lipids leave the cells as triacylglycerol molecules or hydrolyzed products, certainly mechanical processing (mainly grinding) or mastication is necessary for the cells to rupture and allow intracellular lipid and other nutrients (e.g., proteins) to be made available for digestion. Ellis et al. [19] observed the presence of almond tissues (cotyledon and testa) in fecal material after ingestion of almond kernels; some cells were still intact, whereas other cells had partially or totally lost their intracellular lipid. Recently, McArthur and Mattes [21] have subjected masticated samples of almonds, pistachios and walnuts obtained from healthy adults to a static model of gastric and intestinal digestion. While there was no significant difference in the total lipid release between the three nuts after intestinal digestion, walnuts produced a significantly larger particle size after chewing compared with almonds. Furthermore, the particle size after digestion was larger for walnuts compared with pistachios and almonds, indicating additional mechanisms, such as cell wall fissures and lipid storage properties, as relevant for energy extraction from nuts.

#### 2.2.2. Phytochemicals Bioaccessibility

One of the main factors affecting the beneficial potential of polyphenols is their bioaccessibility and absorption in the upper GI tract, followed by their metabolism by the gut microbiota [25]. Polyphenols are a heterogeneous group of compounds characterized by complex structures and polymerization [26]. It is believed that only about 5–10% of the total polyphenol intake could be absorbed in the small intestine, mostly low molecular-weight polyphenols, starting with the removal of the sugar moiety from the glycoside [27]. The chemical structures and associated constituents largely influence their overall absorption, determining whether the polyphenols will be absorbed in the small intestine, or subsequently enter the colon where they could be metabolized by the colonic microbiota. Generally, hydrophobic forces and molecular hindering mechanisms are involved in the in vitro bioaccessibility of lipophilic phenolics, while hydrogen bonding and ionic forces are involved in the bioaccessibility of hydrophilic compounds [28].

We have demonstrated that polyphenols from pistachios are bioaccessible in the upper GI tract, with small differences between raw unsalted and roasted, salted pistachios [29]. It is believed that lutein and zeaxanthin bioavailability from pistachios are enhanced by the presence of fatty acids.

Clinical studies on the bioavailability of almond polyphenols are available. Urpi-Sarda et al. [30] analyzed the polyphenols and their metabolites in the plasma and urine of healthy human subjects after consumption of almond skin polyphenols. Products (O-methyl glucuronide, sulfate, glucuronide and O-methyl sulfate derivatives) of naringenin, (epi)catechin and isorhamnetin were identified in plasma and urine samples in the nanomolar range, together with the glucuronide and sulfate forms of 5-(dihydroxyphenyl)-γ-valerolactone and 5-(hydroxymethoxyphenyl)-γ-valerolactone. Bartolomé et al. [31] identified O-methyl glucuronide, O-methyl sulfate, sulfate and glucuronide derivatives of (epi)catechin, the glucuronide conjugates of isorhamnetin and naringenin, and sulfate conjugates of isorhamnetin, together with conjugates of hydroxyphenylvalerolactones and several products of microbial metabolization in plasma and urine samples. Garrido et al. [32] reported a maximum urinary excretion of naringenin and (epi)catechin conjugates between 2 and 6 h after consumption of almond skin polyphenols, while conjugated metabolites of isorhamnetin and hydroxyphenylvalerolactones reached their maximum levels between 10 and 24 h after consumption.

#### 2.2.3. Effect of Processing and Food Matrix on Digestion

The type of nut and related processing methods greatly influences the damage incurred to the cell wall of the parenchyma, and, thus, the general bioaccessibility, the intracellular diffusion and lipase access to the oil bodies. Verghese et al. [33] have reviewed the effects of processing on the bioavailability of phytochemicals from a range of foods, including nuts, in relation to health benefits of bioactive compounds.

Amongst nut processing methods, dehydration through air or oil roasting can cause microstructural changes, such as lipid coalescence and chemical variations, which affects the integrity and structure of cell walls [34]. Roasting causes textural changes making nut mastication more efficient, which may be explained by the fact that tissues are more brittle when dehydrated. On the other hand, various types of roasting can influence the number of required chews before swallowing [24]. In a recent study, roasting of macadamia nuts changed the appearance of the cell walls and disrupted the oil body membrane, resulting in oil droplet coalescence [35].

Fewer studies have examined the influence of blanching on lipid digestibility, presumably due to the relatively mild process compared with roasting [13]. Oliveira et al. [36] reported that bioactive compounds and antioxidant activities increased with roasting and decreased with blanching. Both processing treatments positively affected the sensorial characteristics, increasing the content of polyunsaturated fatty acids, while saturated fatty acids, monounsaturated fatty acids and several health lipid indices decreased [36].

We have previously reported that incorporating natural and roasted salted pistachios in a food matrix (muffin) decreased the bioaccessibility of certain bioactive compounds, such as protocatechuic acid and luteolin, during in vitro gastric and duodenal digestion [29].

Different food matrices had a significant impact on bioaccessibility of polyphenols from almond skin using a dynamic gastric model. Use of full-fat milk lowered polyphenol recovery, influenced the free total phenols and associated antioxidant status, indicating that phenolics could bind protein within the matrix [37].

A pilot walnut supplementation study of urolithin bioavailability in healthy human volunteers demonstrated that ellagitannin (e.g., punicagalin) metabolism produced a highly variable profile of nine different urolithin metabolites in the urine [38]. Furthermore, the concentration of glucuronidated urolithins in blood and urine did not correlate with antioxidant capacity [39].

Overall, the available literature demonstrated that nutrient and phytochemical release from nuts during digestion is limited and influenced by several factors. Food matrix has an impact on bioaccessibility.

## 3. Effect of Nuts on Gastrointestinal Health

### 3.1. Microbiota Composition and Diversity

Understanding the importance of the gut microbiota (the collection of microorganisms present in a fecal sample) and the gut microbiome (genomes present in the fecal sample) is rapidly advancing. In healthy humans, gut microbiota and microbiome are usually assessed using fecal samples collected after dietary interventions. In these samples, microbial diversity and products of microbial metabolism are typically measured. Microbial end-products of metabolism can also be measured in other biospecimens such as blood or urine. There are many polyphenolic compounds (flavonoids and non-flavonoids) found in nuts. Although these compounds are generally poorly absorbed, they have a wide range of anti-bacterial, anti-inflammatory and anti-carcinogenic effects [40]. These anti-bacterial properties are of interest in how they may affect the host gut microbiota. For example, based on serving size, walnuts are the seventh largest source of total polyphenols among commonly consumed foods and beverages [41,42]. The phenolic profiles and antioxidant activities of free, esterified and bound phenolics in the walnut kernel reveal the presence of a remarkable array of phenolic compounds, including phenolic acids, flavonoids, tannins, phenolic lignans and stilbene-derivatives [43]. The main polyphenol found in walnuts is pedunculagin, an ellagitannin that has a wide range of antioxidant and anti-inflammatory properties [42]. After ingestion, ellagitannins are hydrolyzed to release ellagic acid, which is converted by the gut microflora into the urolithins [42]. With respect to nuts, analyses of microbial diversity, microbiota and microbial end-products have been performed in only a few studies, with more data becoming available as methodologies evolve and analytical costs decrease.

Alpha-diversity is the diversity within a defined microbial community. Typical measures of alpha-diversity are those that account for total species number (species richness) and the relative abundance of species (species evenness). One common measure of species richness is Chao-1, and measures of species richness and evenness include the Shannon index and Simpson index [44]. Chao-1 counts the number of different taxonomic groups (typically genus or species) in a sample, but does not take into account the abundance or relative distributions of the taxa. On the other hand, the Simpson index does consider relative abundance by weighing. Increased alpha-diversity is associated with improved health outcomes [45,46].

Studies of walnuts [47,48], almonds [49,50,51], and pistachios [51] have reported the effect on alpha-diversity of adding these nuts to the diet (intervention type, study design, sample size, dose and study duration are summarized in Table 1). In one study comparing the consumption of almonds or graham crackers as a snack, significant changes were reported in the Chao-1 index and Shannon index [52]. In this study, the snacks were provided for 8 weeks. The authors suggested that the 8 week provision of snacks was longer than many of the other studies, which typically last 3 weeks [47,49,51], and that perhaps these shorter interventions were not of sufficient length to affect alpha-diversity. On the other hand, an additional 8 week intervention of walnuts did not change the Simpson index [53]. Thus, it is unclear what length of feeding is important to affect alpha-diversity, and if tree nut dietary interventions have a substantial effect on alpha-diversity.

Beta-diversity is the diversity among different communities. For some approaches, such as UniFrac distances, qualitative plots are created to show beta-diversity. On the other hand, weighted UniFrac distances are quantitative. Both approaches have been used to measure beta-diversity [44,57]. Increased beta-diversity is associated with improvement in some health outcomes and reduction in BMI.

In four studies of almonds, two studies have reported no effect of almond consumption on beta-diversity using weighted and unweighted UniFrac distances [49,54], one study reported an increase in beta-diversity using unweighted UniFrac distances [51], and one study reported that beta-diversity was measured, but no data were presented [50].

In three studies of walnuts, two studies reported an increase in beta-diversity using weighted principal coordinates analysis of UniFrac distances [47,53]. Additionally, Bamberger et al. also measured beta-diversity with unweighted UniFrac distances, which was also significantly changed with walnut consumption [53]. One study [48] of walnuts used weighted UniFrac distances and did not report significant effects of the walnut diet compared with diets that were matched in fatty acid composition, but did not contain walnuts, or a diet replacing alpha-linolenic acid with oleic acids (also not containing walnuts) after 6 weeks of consuming each diet. The results observed in this study [48] may reflect the similarity of the composition of the diets, and that primarily fatty acid concentrations were manipulated. In one study of pistachios, beta-diversity was reportedly increased [53], and in one study of peanuts, changes in beta-diversity were unchanged between the peanut-containing diet and the control diet [55].

Overall, the effect of nut consumption on alpha- and beta-diversity was inconsistent. Reasons for these reported inconsistencies may be the variability in the length of intervention, the amount of nuts fed, dietary control of the intervention, comparator diets and sample size. The length of intervention for these various studies was 3 to 8 weeks. Since diversity was not a primary outcome, these studies may not have been designed or powered sufficiently to detect changes in diversity. The optimal length of feeding for these types of dietary interventions is unknown. Furthermore, a limited number of studies use provisioned diets which provide all the food consumed by the research volunteers, whereas other studies provide dietary guidance. The latter will likely result in more diet heterogeneity and variability which are two factors that may independently impact microbial diversity. Overall, the amount of nuts offered ranged from 42 to 99 g/d. The differences in these dose levels will likely affect substrate availability for fermentation in the large intestine, given the decreased digestibility of macronutrients in nuts and hence the associated increase in substrate reaching the large intestine [58,59,60,61,62]. Finally, the beta-diversity measured among diets will depend upon the differences in the composition of the diets, and perhaps some of the inconsistencies observed in beta-diversity is a reflection of the similarity of diet comparisons.

#### 3.1.1. Changes in Relative Proportion at the Phyla Level

Changes in the relative abundance of bacteria can be determined at different phylogenetic levels. In a meta-analysis of nut studies, seven interventions investigated phlya-level changes in *Actinobacteria*, *Bacteroidetes*, *Firmicutes*, *Proteobacteria*, and *Verrucomicrobia* [47,49,50,54]. Additionally, two studies reported changes in relative abundance of the phyla *Tenericutes* [50,54]. Additional data were published but were not included in this meta-analysis [8]. Across these interventions, there were no significant effects of nut intake (five almond randomized control trials and one walnut RCT) on the relative abundance of these phlya. In fact, there was only one study in which any of these phyla were altered—with a significant change in the standard mean difference of *Proteobacteria* [50].

#### 3.1.2. Changes in Relative Proportion at the Genus Level

At the genera-level, changes in the relative abundance of 19 phyla have been reported in several interventions [8]. Combining these data, the relative abundance of *Clostridium*, *Dialister*, *Lachnospira*, *Parabacteroides*, and *Roseburia* have been reported [8]. Nut consumption increased the relative abundance of *Clostridium*, *Dialister*, *Lachnospira*, and *Roseburia*. Further nut consumption decreased the relative abundance of *Parabacteroides*. These data represented five almond randomized control trials and one walnut RCT. When the data from the walnut trial were excluded from the meta-analysis, the effect of nut consumption on the relative abundance of *Clostridium* was no longer statistically significant. All of these studies were cross-over designed studies except one, the Dhillon study [54] which was a parallel arm intervention. When the parallel arm study (using almonds) was not included in the meta-analysis, the effect of nut consumption on the relative abundance of *Dialister*, *Lachnospira*, and *Parabacteroides* was no longer significant [8]. For many of these findings, there was heterogeneity, especially related to study duration (studies < 4 weeks vs. studies > 4 weeks), amount of nut consumed (<45 g/d vs. >45 g/d) and nut type (almond vs walnut). While the results of individual studies, and the results obtained from meta-analyses are intriguing, the total number of studies reported is limited, especially when it is likely that overall dose, nut type, and duration of intervention may all affect changes in relative abundance at the genera-level.

### 3.2. Effect of Nuts on Microbial End Products

#### 3.2.1. Short Chain Fatty Acids

Short chain fatty acids (or volatile fatty acids) include acetate, butyrate and propionate. They are a microbial end-product of anerobic fiber fermentation. These short chain fatty acids can be used as an energy substrate by the microbes or host. Short chain fatty acids can block inflammatory processes via activation of G-protein-coupled receptors that are present within colonocytes [63]. These molecular alterations can subsequently activate intracellular signaling pathways that dampen NF-kB activation, modify downstream inflammatory mediators, and increase epithelial barrier function [64]. Species within the genera *Dialister*, *Lachnospira*, and *Roseburia* are known butyrate producers [65]. As mentioned above, the relative abundance of these genera has been shown to be increased with nut consumption.

Specific nut intervention studies that have measured the concentration of fecal short chain fatty acids are limited. In one such study, 87 subjects received 56 g/d of whole almonds or ground almonds (or no almonds as a control) for 4 weeks [18]. Compared with the baseline, there was no change in the fecal concentration of short chain fatty acids (acetate, butyrate, propionate, isobutyrate, valerate or isovalerate) in either the control diet or in subjects consuming either form of almond. However, when the data from the two forms of almonds were combined, there was a higher concentration in fecal butyrate compared with the controls. In a study of 69 subjects also receiving 56 g/d of whole almonds for 8 weeks, there was no observed effect of almond consumption on fecal concentrations of short chain fatty acids [66]. In a study of 63 subjects fed 25 g/d of peanuts, 32 g/d of peanut butter or 32 g/d of control butter made with peanut oil for 6 months, consumption of peanuts and peanut butter increased the fecal concentration of acetate, propionate and butyrate compared with baseline, with no changes in the control group fed butter [56]. In a crossover study of 50 subjects fed 28 g/d of dry roasted, unsalted peanuts or a lower-fat, higher-carbohydrate peanut-free snack for 6 weeks, short chain fatty acids were not measured; however, meta-transcriptomics analysis found that there was an increase in the expression of the bacterial K03518 gene that is directly involved in butyrate production [55]. In a recent short-term study of walnuts, a 3 day consumption in healthy individuals was found to modify the gut microbiome, while also increasing short chain fatty acid levels [67]. Importantly, these effects were dependent upon the composition of the individual microbiome [68]. Walnuts were found to modify the microbiome in an urolithin metabolite-dependent manner. Microbiota analysis further showed significant increases in two bacterial species, namely, *Coprococcus* and *Anaerostipes*, each established producers of butyrate [69]. In addition, *Phascolarbacterium*, a known producer of acetate and propionate, was also increased by walnut consumption [65]. Finally, this study identified significant variability in the metabolism of the polyphenols, differences that were present between the distinct urolithin metabotypes [68].

#### 3.2.2. Bile Acids

The primary bile acids (cholic acid and chenocholic acid) are produced in the liver, while the secondary bile acids (lithocholic acid and deoxycholic acid) are produced in the large intestine by bacterial metabolism. Many bacteria are involved in the conversion of primary to secondary bile acids, including *Bifidobacterium*, *Lactobacillus*, *Clostridium*, *Enterococcus*, *Bacteroides*, *Eubacterium*, and *Escherichia* [70]. The microbially-produced secondary bile acids can bind to nuclear and membrane-bound receptors, activating a complex network of signaling cascades [71,72]. Through these cellular mechanisms, the secondary bile acids have been implicated in various disease etiologies, including several types of cancer, inflammatory bowel disease, cardiovascular disease and non-alcoholic fatty liver disease [72].

In a study of 18 subjects fed 42 g/d of walnuts or an identical control diet without walnuts for 3 weeks, fecal bile acids were measured at the end of each treatment [47]. There were no differences in the concentration of the primary bile acids measured between the two diets. However, after consumption of the diet containing walnuts, the concentration of the secondary bile acids was significantly lower [47]. These walnut-mediated changes in bile acid concentration raise the possibility that walnuts can affect multiple cell-signaling pathways, and possibly disease outcomes, through these microbially-derived end-products [47].

### 3.3. Walnut Consumption and Gastrointestinal Disease

Extensive research has been undertaken to determine whether walnuts may contribute to the mitigation of gastrointestinal disease, particularly with respect to ulcerative colitis and cancer. Walnut constituents contribute to decreased inflammation within the intestinal mucosa, related in part, to the microbial conversion of walnut-derived ellagitannins into a complex family of anti-inflammatory molecules, the urolithins [73]. Of course, walnuts also contain alpha-linolenic acid, a fatty acid that can be readily converted into eicosapentaenoic acid and docosahexaenoic acid, both associated with anti-inflammatory properties [74]. Studies in animal models and in several cell culture systems have uncovered a variety of health benefits that may be attributed to walnuts. A unifying mechanism is likely to involve at least some aspect of effects on immune-related and inflammatory cells. Defining the health benefits of dietary walnut consumption and the influence of its phytochemical composition may stimulate further research into underlying mechanisms that account for disease prevention.

In a preclinical animal model designed to recapitulate the pathology of ulcerative colitis (UC), ellagic acid was found to inhibit disease progression, while reducing associated intestinal inflammation in treated mice [75]. Furthermore, urolithin A, a microbial metabolite of ellagic acid, and its potent synthetic analogue, UAS03, were also found to mitigate DSS-induced intestinal inflammation, with reduced oxidative tissue damage and enhanced intestinal barrier function repair [76]. Both urolithin A and UAS03 provided significant protection against both acute and chronic colitis. This protection was caused by a number of distinct molecular mechanisms, including direct effects on inflammatory mediators, up-regulation of the ligand-activated transcription factor, AhR, and the remarkable ability of these compounds to enhance barrier function by eliciting an up-regulation of claudin 4, a critical tight junction protein [76]. These investigators also evaluated the effects of urolithin A on the direct activation of murine CD4-positive T cells and found a significant repression of their proliferative capacity that was associated with increased miR-10a-5p levels and down-regulation of Orai1/STIM1/STIM2 expression [76]. Koh et al. tested a walnut phenolic extract in both acute and chronic colitis models in mice [77]. This extract was found to inhibit NF-κB signaling, an effect directly associated with reduced expression of pro-inflammatory mediators [78]. Furthermore, Koh et al. also reported that their walnut phenolic extract inhibited colitis-associated colon cancer induced by treatment with the colon carcinogen, azoxymethane, followed by three cycles of 2% DSS for 5 days [77]. Overall, the therapeutic potential of walnuts to positively impact the severity of inflammatory diseases and possibly even inflammation-associated cancer has been established. Finally, Bartoszek et al. have tested the ability of walnut oil to stabilize tight junction proteins and to reduce the levels of pro-inflammatory cytokines commonly present within the inflamed mouse colon following treatment with the ulcerogenic agent, dextran sodium sulfate (DSS) [78]. Promising data from this research group have shown that walnut oil improves overall disease activity and restores normal ion transport and colonic wall permeability [78].

Nakanishi et al. used a similar mouse model to evaluate dietary supplementation with walnuts on colonic mucosal injury induced by DSS [74]. Mice were fed a “Total Western Diet” supplemented with walnuts (ranging from 0 to 14 g walnuts/100 g diet) for two weeks prior to DSS administration. After DSS administration, walnut supplementation significantly protected the colonic mucosa 10 days post-injury. Based on this observed protection against experimentally-induced colitis by walnuts, a follow-up study was conducted to determine the effect of walnuts on metabolites present in the colon [74]. Fecal and colonic samples were analyzed using discovery-based metabolite profiling two weeks post-walnut consumption. Nakanishi et al. found that walnuts caused a significant increase in fecal polyunsaturated fatty acids, including DHA and 9-oxo-10(E),12(E)-octadecadienoic acid (9-oxoODA), as well as kynurenic acid. In the colon, there was a significant increase in S-adenosylhomocysteine and betaine, two important mediators of fatty acid β-oxidation. Together, these findings suggest that metabolic changes caused by walnut consumption may contribute to protection against DSS-induced inflammatory tissue injury [74]. Additional studies are needed to confirm these findings and to better define the precise role of these metabolic changes on colonic inflammation.

Finally, walnut fractions have been found to protect against the development of gastric mucosal lesions, including gastritis, gastric ulcer, and gastric carcinoma [79]. Liu reported gastro-protective and cancer preventive effects of walnut constituents on alcohol-induced inflammation, with fewer gastric lesions and decreased gastric inflammation associated with decreased inflammatory cytokines [80]. Park tested the anti-inflammatory and anti-tumorigenic effects of walnuts in an *H. pylori* gastric cancer model [81]. Mice were infected with *H. pylori* and fed a high-salt diet to promote gastric cancer, and were supplemented with walnuts for nine months. Walnut supplementation caused a significant reduction in gastric cancer frequency with markedly reduced levels of PGE2 and COX-2, important pro-inflammatory mediators that play a key role in tumor promotion [80,81].

Overall, we have demonstrated that, although some nutrients and phytochemicals from nuts are absorbed in the upper GI tract and will reach the colon, clinical studies on their effect on the gut microbiota are still inconclusive. There is literature available on the beneficial effect of walnuts on the prevention of ulcerative colitis and gastric mucosal lesions.

## 4. Conclusions

In the present review, we have outlined the physiological processes that contribute to the digestion of tree nuts. Cell wall composition, thickness and porosity, as well as lipid encapsulation, may slow down or completely prevent some enzymes from entering the cell. It is clear that some fraction of nutrients and phytochemicals present in the nut are not digested in the upper GI tract and could reach the colon, where they may be fermented by the gut microbiota. Although some studies have demonstrated that nut consumption promotes a healthy microbiota, clinical trials are still inconclusive. Importantly, research focused on how nut consumption may affect microbial communities is at an early stage, is further confounded by the wide variability in overall quality of trial design, research methods used, age and health status of subjects, and the amount, type, and duration of nut intake.

## 5. Research Gaps and Future Directions

Future clinical trials must include key measures of microbial community structure, such as species diversity and composition, as well as changes to the microbiome that may be directly related to human health and disease risk. This information will be useful for comparing the beneficial effects of nut consumption across the population. While certain nuts have been investigated more than others for their impact on the GI transit, limited literature is available on the effects of regular consumption of mixed nuts. Research with additional types of nuts is needed to understand their broader effects.

To more accurately assess the health benefits and functionality associated with nut consumption, further studies are needed to better define the mechanisms responsible for their limited energy extraction during digestion, and how the physical structure of individual nuts may ultimately affect bioavailability. Clearly, epidemiological and clinical studies analyzing the potential beneficial effects of nut consumption on prevalent gastrointestinal diseases are warranted in the future.

## Figures and Tables

**Table 1:** Summary of studies evaluating nut intake and microbial changes.

Study Number (REF)	Intervention Nut	Study Design	Sample Size	Dose	Study Duration	Diversity Changes	Microbial Composition Change
[47] (Holscher et al., 2018)	Walnut	Crossover, controlled diet	18	42 g/d	3 wk	No effect on α diversity; β diversity, weighted principal coordinates analysis of UniFrac distances between samples based on their 97% OTU composition and abundances showed that bacterial communities were affected by walnut consumption.	Compared with after the control period, walnut consumption resulted in higher relative abundance of Faecalibacterium, Clostridium, Dialister, and Roseburia and lower relative abundances of Ruminococcus, Dorea, Oscillospira, and Bifidobacterium.
[48] (Tindall et al., 2020)	Walnut	Crossover, controlled diet	42	18% of energy	6 wk	No effect on α diversity; β diversity, weighted principal coordinates analysis of UniFrac distances between samples based on their 97% OTU composition and abundances showed that bacterial communities were affected by walnut consumption.	Compared with after the control period, walnut consumption resulted in higher relative abundance of Faecalibacterium, Clostridium, Dialister, and Roseburia and lower relative abundances of Ruminococcus, Dorea, Oscillospira, and Bifidobacterium.
[49] (Holscher et al., 2018)	Almond (whole, whole roasted, chopped roasted, butter)	Crossover, controlled diet	18	42 g/d	3 wk	No effect on α and β diversity.	Almond consumption increased the relative abundances of *Lachnospira*, *Roseburia*, and *Dialister*. Comparisons between control and the four almond treatments revealed that chopped almonds increased *Lachnospira*, *Roseburia*, and *Oscillospira* compared with the control; whole almonds increased *Dialister* compared with the control. There were no differences between almond butter and the control.
[50] (Burns et al., 2016)	Almond	Crossover, free-living	50	40 g/g	6 wk	No differences in overall microbiota diversity measures (Shannon diversity index and inverse Simpson diversity index).	Targeted qPCR analysis did not show almond intake- associated changes in the quantities of Bifidobacteria spp or lactic acid bacteria. When individual OTUs from 16S rRNA were combined at the phylum level, there were no significant differences in abundances correlating with almond intake. Some changes in the prevalence of various bacterial signatures at the genus and species levels were observed with the almond intervention at final vs. baseline.
[51] (Ukhanova et al., 2014)	Almond	Crossover, controlled diet	18	42 g/d and 84 g/d	18 d	α-diversity was not affected by the intake of almonds.	Numbers of bifidobacteria were not affected by the consumption of almonds.
[51] (Ukhanova et al., 2014)	Pistachio	Crossover, controlled diet	16	42 g/d and 84 g/d	18 d	α-diversity was not affected by the intake of pistachios.	Numbers of bifidobacteria were not affected by the consumption of pistachio. Pistachio consumption appeared to decrease the number of lactic acid bacteria.
[52] (Dhillon et al., 2022)	Almond	Parallel arm, free-living	73	57 g/d	8 wk		Microbial amino acid biosynthesis, and amino sugar and nucleotide sugar metabolism pathways were differentially enriched at the end of the intervention.
[53] (Bamberger et al., 2018)	Walnut	Crossover, free-living	142	43 g/d	4 wk	Supplementing walnuts in the diet did not significantly affect bacterial diversity measured by Shannons effective, and Simpsons effective counts. There was no significant difference in evenness as well as in richness for the walnut diet compared with the control diet. Beta-diversity increased with walnut consumption.	The abundance of Ruminococcaceae and Bifidobacteria increased significantly while Clostridium sp. cluster XIVa species (Blautia; Anaerostipes) decreased significantly during walnut consumption.
[54] (Dhillon et al., 2019)	Almond	Parallel arm, free-living	73	57 g/d	8 wk	Almond snacking resulted in 3% greater quantitative alpha-diversity (Shannon index) and 8% greater qualitative alpha-diversity (Chao1 index) than the cracker group.	Almond snacking decreased overall Bacteroides fragilis relative abundance by 48%.
[55] (Sapp et al., 2022)	Peanut	Crossover, controlled diet	50	28 g/d	6 wk	No between-condition differences in alpha- or beta- diversity were observed.	Following peanut intake, Ruminococcaceae were significantly more abundant compared with a lower-fat higher-carbohydrate snack. Metatranscriptomics showed increased expression of the K03518 (aerobic carbon-monoxide dehydrogenase small subunit) gene following peanut intake, and Roseburia intestinalis L1-82 was identified as a contributor to the increased expression.
[56] (Choo et al., 2021)	Almond	Parallel arm, free-living	69	56 g/d	8 wk	In the almond intervention group, there were significant increases in bacterial community richness, evenness and diversity.	Increases in both the relative and absolute abundance of operational taxonomic units in the Ruminococcaceae family, including Ruminiclostridium, Ruminococcaceae NK4A214, and Ruminococcaceae UCG-003 were the principal drivers of microbiota-level changes.

## Data Availability

Not applicable.
